# The application of artificial intelligence methods to gene expression data for differentiation of uncomplicated and complicated appendicitis in children and adolescents - a proof of concept study –

**DOI:** 10.1186/s12887-021-02735-8

**Published:** 2021-06-08

**Authors:** Josephine Reismann, Natalie Kiss, Marc Reismann

**Affiliations:** Department of Pediatric Surgery, Charité – Universitätsmedizin Berlin, corporate member of Freie Universität Berlin and Humboldt-Universität zu Berlin, Augustenburger Platz 1, 13353 Berlin, Germany

**Keywords:** Appendicitis, Children, Artificial intelligence, Gene expression

## Abstract

**Background:**

Genome wide gene expression analysis has revealed hints for independent immunological pathways underlying the pathophysiologies of phlegmonous (PA) and gangrenous appendicitis (GA). Methods of artificial intelligence (AI) have successfully been applied to routine laboratory and sonographic parameters for differentiation of the inflammatory manifestations. In this study we aimed to apply AI methods to gene expression data to provide evidence for feasibility.

**Methods:**

Modern algorithms from AI were applied to 56.666 gene expression data sets from 13 patients with PA and 16 with GA aged 7–17 years by using resampling methods (bootstrap). Performance with respect to sensitivities and specificities where investigated with receiver operating characteristic (ROC) analysis.

**Results:**

Within the experimental setting a best performing discriminatory biomarker signature consisting of a set of 4 genes could be defined: *ERGIC and golgi 3, regulator of G-protein signaling 2, Rho GTPase activating protein 33, and Golgi Reassembly Stacking Protein 2*. ROC analysis showed a mean area under the curve of 84%.

**Conclusions:**

Gene expression based application of AI methods is feasible and represents a promising approach for future discriminatory diagnostics in children with acute appendicitis.

## Background

Due to new conservative modalities for the treatment of uncomplicated acute appendicitis, current strategies for the distinguishment of high risk and low risk acute appendicitis are under pressure. Successful and safe application of conservative antibiotic treatment for clinically uncomplicated appendicitis has been demonstrated [1]. Even frequent spontaneous resolution of histologically phlegmonous appendicitis has been claimed [2]. However, wrong indication for conservative therapy in cases of underestimated complicated appendicitis can possibly have serious consequences [3].

New evidence on the pathophysiology of acute appendicitis has led to the concept of risk being given more weight without complications having actually (already) occurred. This attitude is especially due to evidence on substantial epidemiological and immunological differences between histopathological phlegmonous and gangrenous appendicitis [4–7]. However, the histopathological level at which complications are more likely to be expected is still matter of debate. Thus, in the current literature, phlegmonous inflammation of the appendix is still considered to be associated with a higher rate of complications [8]. Current studies contradict this view. Phlegmonous (PA) and necrotizing gangrenous appendicitis (GA) are not only characterized by different immunological but also by distinct and time stable laboratory patterns [4–7]. Indeed, the clinically relevant distinctive histopathological level seems to be expected at the stage of necrotic gangrene [9]. In a recent study hyponatremia has turned out as a promising parameter for delineation of perforated appendicitis [10]. A high decisive degree for the diagnosis of perforation can also be reached with advanced combinatory tools as the appendicitis inflammatory response (AIR) score [11]. However, clear demarcation of appendicitis at the earlier stage of non-perforated gangrene would even expand the scope for therapeutic action with the aim to avoid the most severe complications.

In a prospective study with genome wide gene expression analysis in children with acute appendicitis showed clearly distinguishable pictures within an immunological pathway analysis [12]. Gene expression patterns in patients with phlegmonous appendicitis were highly suspicious for activation of antiviral immunological mechanisms, while patterns in patients affected by gangrenous inflammation can be generally characterized as antibacterial.

However, safe differentiation for clinical decision making is still a great challenge. Clinical examination is characterized by a great interobserver variability with low discriminatory capacity [13], laboratory values vary too widely for safe differentiation [6, 13] and imaging techniques like ultrasound show promising results with respect to the significance of particular parameters, but are dependent on the expertise of and interpretation by the investigator [14].

In a recently published study, laboratory cellular parameters from full blood counts, C-reactive Protein (CRP) and the sonographically measured appendiceal diameter were used within modern algorithms from machine learning and artificial intelligence for the differentiation of phlegmonous and gangrenous appendicitis [15]. The predictive capacity could be substantially improved compared with that of the single parameters in this experimental setting.

The aim of the current study was to investigate the applicability of algorithms from machine learning and artificial intelligence to the extended pool of data from whole genome gene expression analysis for pretherapeutic differentiation of phlegmonous and gangrenous appendicitis in children and adolescents.

## Methods

This prospective study included children aged 7–17 years, which presented with signs of acute appendicitis at the Department of Pediatric Surgery of Charité – Universitätsmedizin Berlin, Germany, between April 2019 and August 2019. Ethical approval was provided by the local ethics committee (reference number ES2/130/16). Due to ethical concerns regarding a gene expression study with underaged patients, the study population was restricted to a total number of 30 patients with an additional expected exclusion rate of 10%. The ability to give informed consent based on age adapted information sheets within this pilot study was expected at an age of 7 years. Thus, younger children were not considered. Patients with signs of acute appendicitis in ultrasound examination and planned appendectomy were enrolled in the study using respective information sheets for informed consent (separate leaflets for children aged 7–14, for children aged 15–17 years and for parents). Inclusion criteria were written informed consent, performed appendectomy with histopathological examination, sufficient RNA quality (RNA integrity number over 7) and time period from blood sample collection till peripheral blood mononuclear cell (PBMC) isolation less than 1 h. Exclusion criteria were previous conservative treatment of acute appendicitis, any concomitant disease or antibiotic treatment in the past 2 weeks.

All patients underwent standard laparoscopic appendectomy in general anesthesia with infraumbilical insertion of a 12 mm Hasson trocar for a 5 mm camera and of two 5 mm working trocars in the left and mid lower abdomen for 5 mm instruments. After establishment of a capnoperitoneum (10-12 mmHg) and bipolar dissection of the appendicular artery, the appendix was ligated with Röder-slings at its base, divided with scissors and removed via retrieval bag. A 5 mm stapler device was used for complicated appendicitis with necrotic appendix base. In case of free fluid irrigation with Ringer solution and respective suctioning was performed. Drains were consequently avoided.

### Isolation of PBMCs and RNA

According to the ethical vote, blood samples (> 5 ml) were only collected during routine blood test at the emergency department. Isolation of PBMCs was carried out within 1 h after blood collection. Therefore, blood from each patient was suspended in phosphate-buffered saline (PBS, 1:1 ratio) followed by density gradient centrifugation (Ficoll PM400, GE Healthcare, Pittsburgh, PA; room temperature, 30 min at 400 g). Thereafter, the monocyte layer was re-suspended with PBS and centrifugated (twice, 5 min each at 400 g), followed by a final re-suspension with 1 ml PBS, centrifugation and removal of the supernatant. Native cells were frozen at − 20 °C (Mr. Frosty, Waltham, MA) and finally stored at − 80 °C in liquid nitrogen. By using the NucleoSpin RNA Plus kit (Macherey-Nagel, Germany) total RNA was isolated from the PBMCs. RNA quality control (2100 Bioanalyzer, Agilent Technologies; RNA 6000 Pico Kit) and RNA quantity control (Nanodrop 2000 spectrophotometer; Thermo Scientific) were performed. Samples displaying a Bioanalyzer’s RNA integrity number (RIN) above seven fulfilled quality control standards and were labelled by generation of fluorescent cRNA (complementary RNA; Low Input QuickAmp Labeling Kit, Agilent Technologies). 1st strand synthesis using random primer/oligo-dT primer mixture, 2nd strand synthesis and synthesis of cRNA labelled with cyanine 3-CTP and were performed. Cy3 labelled cRNA (600 ng) was hybridized (65 °C for 17 h; ArrayXS Human Agilent microarray; design ID 79407; Agilent Gene Expression Hybridization Kit, Agilent Technologies; Oak Labs, GmbH, Henningsdorf, Germany) followed by microarray wash and scanning (SureScan Microarray Scanner; Agilent Technologies). According to manufacture’s protocols a resolution of 3-μm was used to generate 20 bit TIF files.

### Histopathological examination

After appendectomy, the appendices were histopathologically examined by two pathologists of which the second examination was a blinded evaluation by a specialized pediatric pathologist. Thereafter, primary diagnosis had been corrected in two cases. Appendicitis was classified in accordance with Carr (9):

- phlegmonous appendicitis: transmural infiltration of the appendix by neutrophilic granulocytes, serositis, microabscesses and oedema without gangrene or perforation
- gangrenous appendicitis: ischemic areas in the appendix with transmural myonecrosis.
- perforated appendicitis: gangrenous alterations with transmural defect of the appendix wall

### Microarray analysis

To obtain microarray data, the Feature Extraction Software V11 (Agilent Technologies) and GE1_1105_Oct12 protocol was used to extract TIF files. At OakLabs GmbH, Henningsdorf, Germany, the raw data was further analyzed using DirectArray Software. Box plots were used to visualize signal distributions of raw data and to identify potential issues for individual samples. For statistical analysis of gangrenous versus perforated appendicitis, samples were first quantile normalized and then analyzed using a Welch’s test and calculating log2 fold changes for each gene. The level of significance was *p* < 0.05.

### Development of biomarker signatures for differentiation of phlegmonous and gangrenous appendicitis

A supervised learning algorithm was used to analyze gene expression data and to build a prediction model for diagnosis of appendicitis based on relevant biomarkers. Usually, this is a two-step process summarized as *discovery* and *validation *[15]. Due to a limited number of included patients, we favored the widely accepted resampling technique to alternate between the discovery and the validation phase in up to thousand iterations. Thus, to develop biomarkers in this experimental setting, resampling was performed with the bootstrap method for dataset augmentation: Input data consisted of a large number of subsets of samples for varying threshold values. Model building with biomarker selection was carried out on a portion of these data (“discovery set”) with measurement of the performance in an independent data set (“validation set”). Input data consisted of *n* samples, each of these described by a set of *p* variables – represented by the biomarker values. Concretely, the data matrix consisted of *n* lines and *p* columns. Then, relevant biomarkers were identified: a sequence of distinct biomarker signatures {bm_1_,. . ..,bm_j_,.. ., bm_m_} was built and subsequent implementation of a binary classification problem was performed, fitting the parameters of a logistic regression model on the discovery data X_discovery_ whose colums *p*_*bmj*_ were filtered according to the biomarker signatures. The quality of each biomarker signature was measured on the discovery data, while all performance values were obtained by measurement of the trained model on the validation data [15].

After definition of the best model, it was used to predict the diagnostic status of a patient with class probability. Output class probabilities can be interpreted as separation thresholds between class prediction. The thresholds represent trade-offs for the model to predict true/false positive and true/false negative rates. The diagnostic ability of the model with regard to sensitivities and specificities was tested on the validation dataset: true and false positive rates were counted at different thresholds [15]. For illustration of the results, a receiver operating characteristic (ROC) plot was created.

The primary endpoint of the study was the pretherapeutic distinctness of the histopathological entities demonstrated by an area under the curve (AUC) in the ROC analysis exceeding at least 50%. A relevant decisive degree was assumed at an AUC of at least 80%.

### Statistical analysis

Regarding evaluation of the artificial intelligence approach concerning effective differentiation of the inflammatory entities, bootstrapping allowed for the calculation of standard errors and confidence intervals.

Statistical analysis regarding epidemiological and routine laboratory data was performed with Mann-Whitney-U-test for continuous and Chi-Square-test for categorical parameters. Welch’s t-test was used for statistical analysis of gene expression values after quantile normalization as previously described [10]. Values are shown as percentages or mean ± SD. Level of significance was *p* < 0.05. Statistical analysis was performed with GraphPad Prism software (version 9.1.0, La Jolla, CA).

## Results

### Patients and samples

Epidemiological data have already been published elsewhere [12]. Acute appendicitis was suspected sonographically in 33 otherwise healthy patients. After primary inclusion following informed consent, four cell samples (three samples of patients with GA, one of a patient with PA) were secondarily excluded due to signs of degradation within the RNA quality control. After two histopathological examinations the PA group consisted of 13 and the GA group of 16 samples. Mean age in the PA group was 11.5 ± 2.7 years and in the GA group 11.1 ± 2.6 years. Gender distribution was as follows: 4 patients in the PA group were female and 9 male, in the GA group 10 patients were female and 6 male. Mean CRP was significantly upregulated in patients with GA compared with those affected by PA (71.4 ± 58.81 mg/L vs. 23.05 ± 19.26 mg/L, *p* < 0.05). Mean eosinophilic granulocytes were significantly upregulated in patients with PA compared with patients with GA (0.8 ± 0.1 vs. 0.6 ± 0.18, p < 0.05) without any other significant differences in the differential blood counts.

Patient demographics, distribution of histopathological entities and mean symptom durations are illustrated in Table 1.

**Table 1:** Epidemiological data and sympton duration until sample collection

	total (***n*** = 29)	phlegmonous (***n*** = 13)	gangrenous (***n*** = 16)
**Mean age (years)**	11.3 ± 2.6	11.5 ± 2.7	11.1 ± 2.6
**Gender**			
**Female**	14 (48%)	4 (31%)	10 (62.5%)
**Male**	15 (52%)	9 (69%)	6 (37.5%)
**Duration of symptoms (hours)**	28.3 ± 16.9	24 ± 13.3	32.1 ± 19.4

Mean values ± SD or total numbers and percentages

### Biomarker signatures for differentiation of phlegmonous and gangrenous appendicitis

**Fig. 1:**
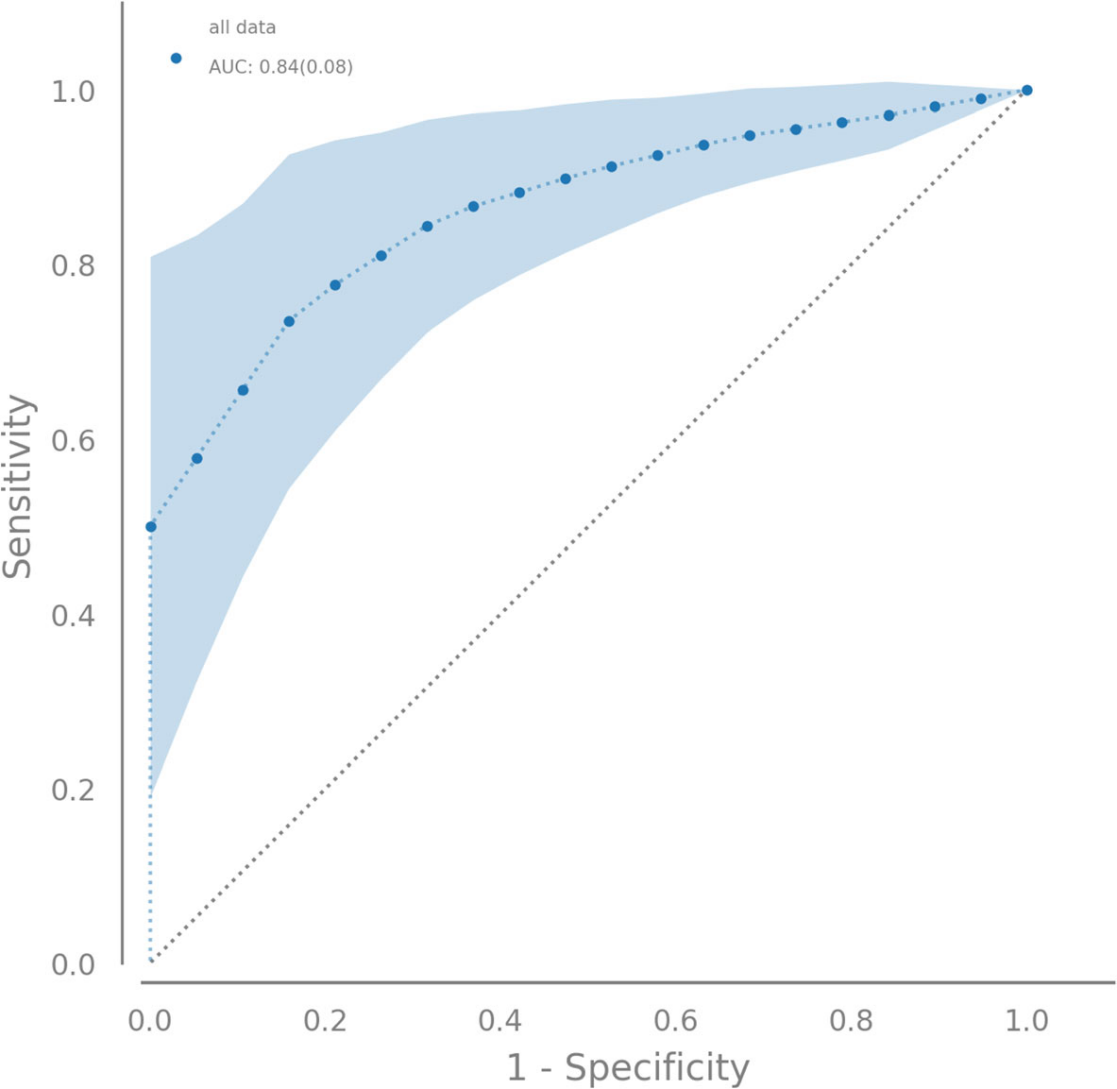
Receiver operating characteristic curve after application of resampling with varying thresholds. Biomarker consisting of a set of 4 genes. *ERGIC and golgi 3, regulator of G-protein signaling 2, Rho GTPase activating protein 33, Golgi Reassembly Stacking Protein 2;* mean values ± SE

As published previously, out of a total of 56,666 analyzed genes 3594 (6.3%) were significantly differentially expressed [12]. Resampling allowed the definition of a best performing discriminatory biomarker signature which consisted of a set of four genes from of a total of 56,666 genes: *ERGIC and golgi 3, regulator of G-protein signaling 2, Rho GTPase activating protein 33, and Golgi Reassembly Stacking Protein 2*. Figure 1 displays the receiver operating characteristic curve with illustration of the particular sensitivities and specificities of the analyzed thresholds after resampling (mean values ± SE). The area under the curve (AUC) was 84% (SE 8, CL 68.2).

## Discussion

Reliable differentiation of the inflammatory entities within acute appendicitis is mandatory for safe performance of new conservative treatment strategies. Several studies have attempted to increase the validity of currently established routine methods with regard to the pretherapeutic differentiation between complicated and uncomplicated appendicitis. But especially the great number of publications which are concerned with this question might be a sign for the fact that this problem is still unsolved.

In particular, we identified two obstacles in previous attempts to resolve this issue. First, up to now the level at which the distinction should be made has not been convincingly shown – at the level of clinical judgement or at that of histopathological examination. And second: The discriminatory capacity of the established diagnostic methods is far too low with respect to the needed sensitivities and specificities. Clinical examination and imaging techniques are severely limited by interobserver reliability and need for interpretation, currently used laboratory values are restricted by a substantial indistinctness [6, 13, 14].

Increasing epidemiological, immunological and even gene expression evidence strongly suggests that acute appendicitis should be differentiated with respect to expected courses (uncomplicated vs. complicated) at the histopathological levels of phlegmone and gangrene. Phlegmonous appendicitis is most probably an own entity based on a distinct pathophysiology, possibly related to viral infection [4, 5, 12]. In contrast, gangrenous appendicitis is an own necrotic manifestation with strong immunological signs of bacterial origin [4, 5, 12]. Although clinically hardly distinguishable over a period of time, gangrenous inflammation comprises a substantially increased risk for complicated courses, while phlegmonous disease is comparably harmless and can be treated conservatively or even resolves by itself [2, 7, 16]. In order to provide best care for patients - in our case affected children - the distinguishment should be already effective at an early stage at which the entities might eventually not yet be distinguishable with current means.

Application of supervised learning algorithms to gene expression data with focus on targets for therapeutic intervention and biomarkers for early diagnosis has already been successfully performed in other contexts like sepsis [17]. It was previously shown that methods of machine learning and artificial intelligence can be used to substantially improve the significance of routine inflammatory values and the sonographically measured appendix diameter within biomarker signatures for the differentiation of histologically phlegmonous and gangrenous appendicitis in children [15]. In the herewith presented approach the experimental biomarker signature with a total of 29 patients reached an AUC of 84% - even slightly outperforming that of the previously published application of artificial intelligence to routine values with its 590 included patients (AUC 80%) [15].

Interestingly, the particular underlying expressions of the particular genes in the biomarker signature (*ERGIC and golgi 3, regulator of G-protein signaling 2, Rho GTPase activating protein 33, Golgi Reassembly Stacking Protein 2*) are involved in cellular functions affecting cytoskeleton formation, regulation of GTP binding, membrane trafficking and cell signalling [18]. Unlike the other included genes, *ERGIC and golgi 3* is not significantly differentially regulated (*p* > 0.05). This finding demonstrates that the diagnostic horizon, which is usually limited to standard parameters when purely statistical methods are used, is expanded through the use of artificial intelligence. However, as these cellular functions take place on a very basic level, interpretation is not easy and cannot be provided at this stage.

The comparability of the present approach with other studies is limited. It represents a purely experimental procedure in terms of a proof-of-concept study with a limited number of patients.

## Conclusion

Although the presented investigation is best characterized as a simulation with artificial augmentation of data sets, the results justify the application of supervised learning algorithms with regular training and validation sets to gene expression data of a greater number of patients in order to gain the necessary level of distinguishability of the inflammatory entities. Although the presented results are already very promising, the combination of gene expression data with other largely objective parameters like the sonographically measured appendiceal diameter within artificial intelligence strategies might even substantially improve the efficacy of the method.

## Data Availability

The datasets generated and analyzed during the current study are available in the Open Science framework repository (www.osf.io) under the URL https://osf.io/xwnr3/.

## Abbreviations

AI: Artificial intelligence
PA: Phlegmonous appendicitis
GA: Gangrenous appendicitis
CRP: C-reactive protein
RNA: Ribonucleic acid
PBMC: Peripheral blood mononuclear cell
ROC: Receiver operating characteristic
